# Supramolecular Solid Complexes between Bis-pyridinium-4-oxime and Distinctive Cyanoiron Platforms

**DOI:** 10.3390/molecules29081698

**Published:** 2024-04-09

**Authors:** Igor Picek, Dubravka Matković-Čalogović, Goran Dražić, Gregor Kapun, Primož Šket, Jasminka Popović, Blaženka Foretić

**Affiliations:** 1 Department of Chemistry and Biochemistry, School of Medicine, University of Zagreb, Šalata 3, HR-10000 Zagreb, Croatia; ipicek@mef.hr; 2 Department of Chemistry, Faculty of Science, University of Zagreb, Horvatovac 102a, HR-10000 Zagreb, Croatia; dubravka@chem.pmf.hr; 3 National Institute of Chemistry, Hajdrihova 19, SLO-1001 Ljubljana, Slovenia; goran.drazic@ki.si (G.D.); gregor.kapun@ki.si (G.K.); primoz.sket@ki.si (P.Š.); 4 Division of Materials Physics, Ruđer Bošković Institute, Bijenička Cesta 54, HR-10000 Zagreb, Croatia

**Keywords:** pyridinium oxime, hexacyanoferrate(II), nitroprusside, charge-transfer complexes, supramolecular complexes, optical materials, cyanoiron complexes

## Abstract

The structural features and optical properties of supramolecular cyanoiron salts containing bis-pyridinium-4-oxime Toxogonin^®^ (TOXO) as an electron acceptor are presented. The properties of the new TOXO-based cyanoiron materials were probed by employing two cyanoiron platforms: hexacyanoferrate(II), [Fe(CN)_6_]^4–^ (HCF); and nitroprusside, [Fe(CN)_5_(NO)]^2–^ (NP). Two water-insoluble inter-ionic donor–acceptor phases were characterized: the as-prepared microcrystalline reddish-brown (TOXO)_2_[Fe(CN)_6_]·8H_2_O (**1a**) with a medium-responsive, hydrochromic character; and the dark violet crystalline (TOXO)_2_[Fe(CN)_6_]·3.5H_2_O (**1cr**). Complex **1a**, upon external stimulation, transforms to the violet anhydrous phase (TOXO)_2_[Fe(CN)_6_] (**1b**), which upon water uptake transforms back to **1a**. Using the NP platform resulted in the water-insoluble crystalline salt TOXO[Fe(CN)_5_(NO)]·2H_2_O (**2**). The structures of **1cr** and **2**, solved by single-crystal X-ray diffraction, along with a comparative spectroscopic (UV–vis–NIR diffuse reflectance, IR, solid-state MAS-NMR, Mössbauer), thermal, powder X-ray diffraction, and microscopic analysis (SEM, TEM) of the isolated materials, provided insight for the supramolecular binding, electron-accepting, and H-bonding capabilities of TOXO in the self-assembly of these functionalized materials.

## 1. Introduction

**Scheme 1:**
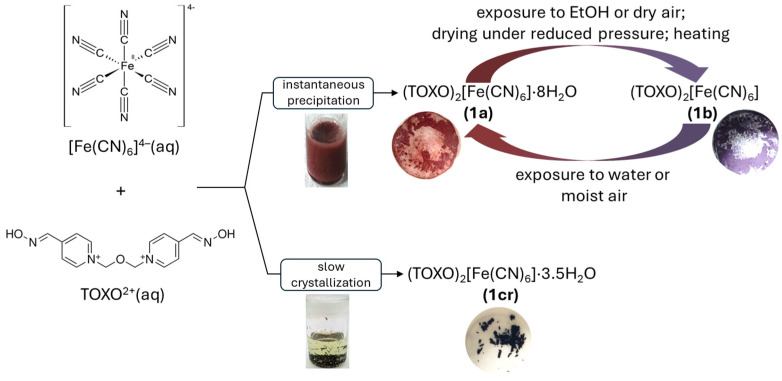
Isolation and hydrochromic properties of supramolecular (TOXO)_2_[Fe(CN)_6_]·nH_2_O solids.

Toxogonin^®^ (TOXO), also known as obidoxime (see Scheme 1 for structural formula) belongs to the group of pyridinium-4-oximes available in medical practice as the real antidote in organophosphorus poisoning (pesticides, nerve gases) [1,2]. Organophosphorus toxicity elicits a broad spectrum of physiological responses, and inhibition of the enzyme acetylcholinesterase (AcChE) certainly plays an important role [3,4]. Inhibition of AcChE occurs with resultant excessive stimulation of the nervous system, leading to respiratory failure and, in some cases, death. A variety of acute responses can take place in the categories of muscarinic and nicotinic features [3]. Pyridinium oximes, powerful nucleophiles, act as esterolytic agents used in the cleavage of ester or amide bonds [5]. Furthermore, the carbonyl derivative of pyridinium-4-oxime plays an important role in organic synthesis as an excellent Michael donor in nucleophilic additions [6]. Their pharmacological role is mainly a result of nucleophilic reactivation of inhibited AcChE and restoration of enzyme activity. Consequently, pyridinium oximes can mitigate muscarinic (nausea, vomiting, heart block, pulmonary edema, and abdominal cramps) and nicotinic (hypertension, weakness, and muscle fasciculation) effects of poisoning [3,7]. The overall interaction of the phosphorylated enzyme and reactivators is complex and highly dependent on the chemical structure of the inhibitor, type of phosphorus residue at the enzyme’s active center, structure of the pyridinium oxime compound, and conformational and spatial interactions between active groups of the reactivators and phosphorus residue in the enzyme molecule [7]. Moreover, among other factors contributing to their bioactivity, the electrochemical properties of pyridinium oximes are important since if the reduction potential is more positive than −0.5 V, then electron transfer (ET) is a possible process in vivo; this is an active research topic [3,4]. The enhanced bioactivity of bis-pyridinium salts, like TOXO itself, has been attributed to improved binding orientation, but from the electrochemical viewpoint, a second electroactive pyridinium-4-oxime moiety might elicit an enhanced electrical response [3]. Focused on electron- and charge-transfer processes, pyridinium-4-oxime cations have recently been recognized as new electron acceptors for the formation of colored, supramolecular, inter-ionic charge-transfer complexes, with the hexacyanoferrate(II) anion as a donor [8]. The use of highly charged cyanoiron donors and *N*-heterocyclic mono- and dications to build supramolecular charge-transfer complexes has attracted much attention, especially with the 4,4′-dipyridinium dication (viologen) and its derivatives [9]. The inter-ionic charge transfer in such systems resembles the intervalence charge-transfer of Prussian blue and Prussian blue analogues, important in construction of novel inorganic/organic hybrid polymeric materials. In particular, metal–organic frameworks have been able to unite physical and chemical properties of different organic/inorganic components for many applications [10,11], including catalysis [12], separations [13], magnetism [14], sensing [15], and gas storage [16]. In addition, it is well known that complexation of biologically active compounds, such as TOXO, can be applied as an efficient method to improve their initial properties via various mechanisms. Moreover, interest in metal–nitrosyl complexes has increased, especially in those that release nitric oxide (NO), nowadays a well-recognized signaling molecule in important biological processes (i.e., blood pressure regulation, wound healing, memory formation) [17]. Pharmacological use of sodium nitroprusside (pentacyanonitrosylferrate) has been known since 1950, acting as a powerful vasodilator and one of the major nitric NO releasers available. The physico-chemical mechanism for release of NO by nitroprusside has been widely investigated and recently reviewed [18]. The mechanism is complex since it can generate multiple species such as NO, CN^−^, Prussian blue, and in certain cases even HNO. Photoexcitation of metal–nitrosyl complexes leads to NO release or isomerism of the NO linkage, and its photosensitivity depends mainly on the energy of the dpπ(M) → π*(NO) metal-to-ligand charge transfer (MLCT) transition [19].

The aim of this work is to provide insight into the supramolecular binding, electron-accepting, and H-bonding capabilities of TOXO in solid cyanoiron materials. A recent study of pyridinium-4-oximes revealed that the bis-pyridinium-4-oxime dication, TOXO, had the highest electron-accepting ability in an aqueous medium [8]. To characterize its electron-acceptor properties and self-assembly potential in the solid state and to test the different but related effects of the employed electron donor, compared to hexacyanoferrate(II) [Fe(CN)_6_]^4–^ (HCF), the nitroprusside [Fe(CN)_5_(NO)]^2−^ (NP) unit, with a similar octahedral geometry but different electronic properties, has been used here. Herein, by comparative spectroscopic (UV–vis–NIR diffuse reflectance, IR, solid-state MAS-NMR, Mössbauer), thermal, and crystallographic (SCXRD and XRPD) studies the exciting properties of these new supramolecular materials are presented.

## 2. Results and Discussion

By using HCF and NP platforms, four supramolecular hybrid salts were isolated, as the use of HCF resulted in the formation of three distinct phases: two microcrystalline hydrochromic powders with significantly different water content, and one single-crystal phase. The colors of the isolated solids (TOXO)_2_[Fe(CN)_6_]·nH_2_O (**1**), where n = 0, 3.5, or 8, which are not combinations of the constituents, imply an inter-ionic charge transfer (IICT) from HCF to TOXO. In contrast, the self-assembly of TOXO and NP gave a new organic-hybrid salt, (TOXO)[Fe(CN)_5_(NO)]·2H_2_O (**2**), in which TOXO is present solely as a counterion.

### 2.1. Inter-Ionic Charge-Transfer Hexacynoferrate(II) Supramolecular Structures

For this study we mainly employed previously described procedures for the isolation of fresh supramolecular solids [8]. When aqueous solutions of HCF (donor) and TOXO (acceptor) were mixed, and the concentration of the components exceeded 2 mM at RT, a microcrystalline reddish brown solid (**1a**) instantaneously precipitated. After the precipitate was filtered off and washed with deionized water, even drying in air resulted in the color change from reddish-brown to violet, previously identified as anhydrous (TOXO)_2_[Fe(CN)_6_] (**1b**). Now, this material was found to be sensitive to water and hydration produced a reddish-brown, microcrystalline powder (**1a**). These changes could be easily observed by the naked eye. Trying to obtain SCXRD-quality crystals, we used stable hexacyanoferric(II) acid (H_4_[Fe(CN)_6_]) as a source of HCF since numerous crystalline structures of organic hexacyanoferrates, including the pyridinium-type cations, have been prepared and described [20,21,22,23,24]. Many such complexes contain protonated cyanoferrate(II) platforms, and therefore, regardless of the charge of the organic cation, they have different molecular formulas, determined by the different charge of the cyanoferrate(II). Our results show that regardless of the source of HCF and the single-crystal preparation technique, as well as the molar ratio of the donor and acceptor, the rapid formation and precipitation of the IICT complex with a composition of 2 TOXO to 1 HCF occurs. By using a 3 M KCl reaction solution, the increased ionic strength slowed down the precipitation, allowing formation of dark violet SCXRD-quality crystals (**1cr**). The SCXRD analysis revealed the unique supramolecular structure, in which the molar ratio of TOXO to HCF was also 2:1, but without observable hydrochromic properties. The isolation of all three phases and the observed hydrochromic behavior is presented in Scheme 1.

Interestingly, all three phases significantly differ in water content, as evidenced by the thermal TG/DSC/DTA analysis. The TGA profiles (shown in Figure S1) indicate, for the hydrated phase **1a**, a weight loss of 15.45%, which corresponds to the evolution of eight water molecules per chemical formula unit (calculated 15.90%) and occurs at the first step in the temperature range up to 100 °C, while the anhydrous phase **1b** reveals no significant weight change. Identical shapes for the TG curves of **1a** and **1b** are observed upon further heating up to 500 °C. In the case of the crystalline phase **1cr**, the weight loss of 7.30% per chemical formula unit (calculated 7.40%) that corresponds to the loss of three and half water molecules is observed in the temperature range up to 160 °C. These results indicate that water molecules within **1a** are significantly more loosely bounded than in **1cr**. Then, higher thermal energy, in the range of 190 to 210 °C, leads to analogous well-defined exothermic weight loss, characteristic for cyanoiron(II) complexes [25], revealing their analogous composition to (TOXO)_2_[Fe(CN)_6_], which continues to decompose on further heating. These gravimetric results show that the change in color between **1a** and **1b** is accompanied by extrication of eight water molecules per chemical formula unit. In addition, the hydrated reddish-brown **1a** transforms to anhydrous violet **1b** on drying at room temperature or under reduced pressure and upon exposure to ethanol. The obtained **1b** transforms back to **1a** upon exposure to water or humidification. 

**Figure 1:**
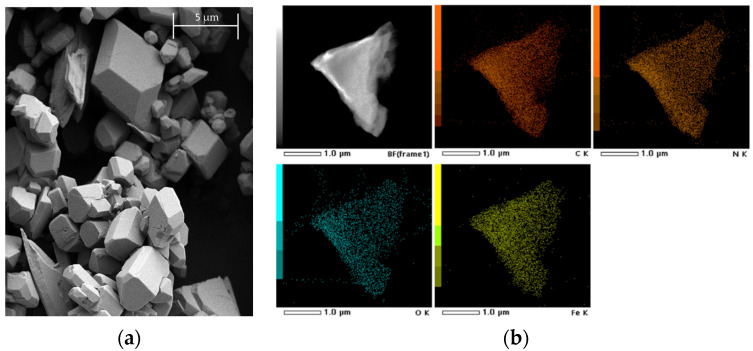
(**a**) SEM image of **1b**; (**b**) energy-dispersive X-ray spectroscopy (EDXS) elemental maps.

An SEM image of **1b** is shown in Figure 1a, while the STEM analysis revealed that the selected fragments were uniform, free of visible defects or secondary phases, and composed of carbon, nitrogen, oxygen, and iron, as determined by energy-dispersive X-ray spectroscopy (EDXS), shown in Figure 1b. The mean value of the iron mass percent of 7.2, estimated by EDXS, agrees with the calculated mass percent of 7.1.

**Figure 2:**
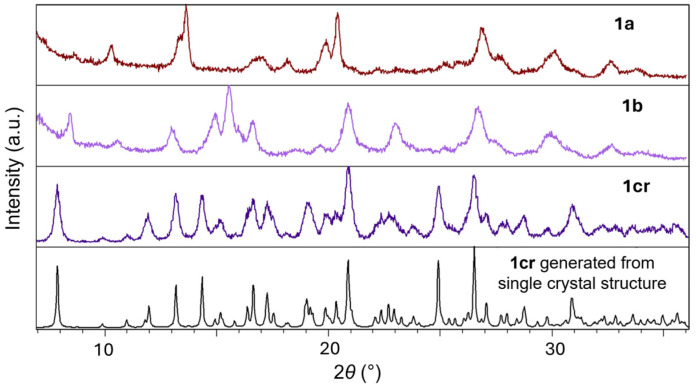
Experimental XRPD patterns of hydrated phase **1a** as-prepared, anhydrous phase **1b,** and crystalline phase **1cr**, respectively. Bottom tab shows simulated XRPD pattern calculated from the single-crystal structure of **1cr**.

The XRPD pattern of the reddish-brown **1a** is different to that of the violet **1b** and substantially distinct from **1cr** (Figure 2). The similar breadths of the diffraction lines of the hydrated **1a** and anhydrous **1b** indicate that there is no significant loss of crystallinity upon dehydration.

**Figure 3:**
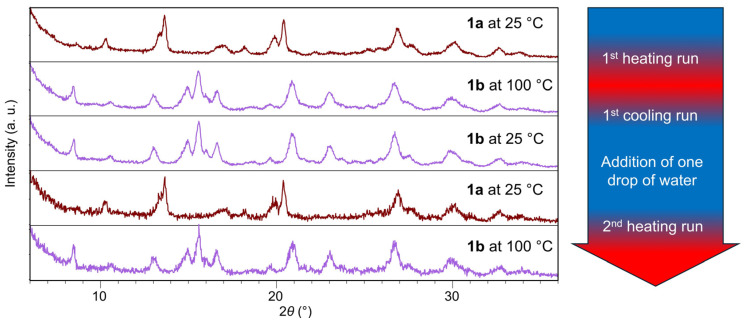
Two cycles of in situ variable-temperature XRPD measurements, showing the temperature-induced release of water molecules from **1a** phase resulting in the formation of **1b** anhydrous phase that, upon water exposure, transforms back to **1a**.

To evidence the reversible hydrochromic structural transformation between **1a** and **1b**, a time-dependent in situ XRPD analysis was carried out (Figure 3). Figure 3 shows that **1a** undergoes phase transition to anhydrous **1b** upon heating at 100 °C. The anhydrous structure remains unchanged during cooling to RT. Upon addition of one drop of water to anhydrous **1b**, the crystal structure of **1a** recovered instantly, confirming the reversibility of the water-induced structural changes and preservation of structural integrity. The release of water molecules was again observed during the second heating run. The XRPD pattern of the regained reddish-brown **1a** is in excellent agreement with that of the as-prepared form **1a**. The hydration–dehydration cycle causes reversible structural changes that induce a shift in the inter-ionic CT interactions responsible for the color of these self-assembled hydrochromic compounds.

**Figure 4:**
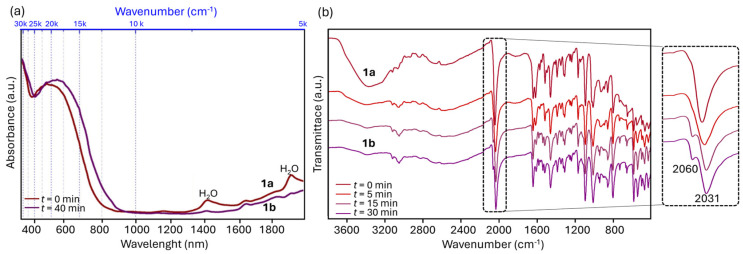
(**a**) Time-dependent in situ changes in the UV–vis–NIR diffuse reflectance spectrum of **1a** upon standing and drying at room temperature, i.e., transforming into **1b**; (**b**) time-dependent in situ ATR-FTIR spectra showing the release of water molecules from **1a**, resulting in the formation of anhydrous **1b**.

The reversible hydrochromic character of **1a** and **1b** at ambient temperature, pressure, and humidity is evidenced by in situ, time-dependent UV–vis–NIR diffuse reflectance spectroscopy. The spectra are presented in Figure 4a, showing a notable bathochromic shift of the IICT band maximum originating from the d(t_2g_^6^)p(π^0^)^*^→d(t_2g_^5^)p(π^1^)^*^ transition centered at 485 nm to 548 nm (Δ*λ* = 63 nm) upon dehydration of the reddish-brown **1a,** and its spontaneous transformation to the violet **1b**. At the same time, bands in the NIR region assigned to water molecules disappear. The obtained values of optical gap energies (*E*_gap_), representing the energy of transition from the ground molecular state to the lowest excited state, for **1a** (1.84 eV) and **1b** (1.76 eV [8]) further suggest that each phase exhibits different inter-ionic CT interactions. The observed decrease in energy of the IICT band as well as the *E*_gap_ upon dehydration reflects structural perturbations that lead to better overlapping of the donor’s HOMO d(t_2g_^6^) and the acceptor’s LUMO p(π^0^)* orbitals, which enhances the inter-ionic CT interaction in **1b**. The spectrum of the dark violet **1cr** (Figure S2) shows a very broad IICT band, with the maximum centered around 550 nm. Interestingly, the UV–vis segment of the spectrum is like anhydrous **1b** and substantially different to **1a**, emphasizing the role of water molecules in the supramolecular charge-transfer assembly. Presumably, the higher content of water present in **1a** increases the distance between HCF as donor and TOXO as acceptor, resulting in higher energy being needed for the d(t_2g_^6^)p(π^0^)^*^→d(t_2g_^5^)p(π^1^)^*^ transition.

Considering the possibility for the TOXO moiety to form the radical via a one-electron transfer from HCF, we performed ^57^Fe Mössbauer measurements at RT and 80 K. The ^57^Fe Mössbauer parameters for **1b**, isomer shift (IS), and a quite small quadrupole splitting (QS) are typical values for low-spin iron(II) complexes, while the subtle QS value seems to be attributable to the slightly distorted octahedral environment around the iron(II) ions (Figure S3). These values are comparable with those found for K_4_[Fe(CN)_6_]·3H_2_O itself and other HCF-based materials, without a notable effect of the zeolitic or crystallization water on their ^57^Fe Mössbauer spectra [26]. The ^57^Fe Mössbauer study shows that, in both **1a** and **1b**, there is no quantifiable degree of electron transfer (ET), even at 80 K. Namely, ET would cause charge separation and formation of [Fe^III^(CN)_6_]^3–^ and pyridinyl radicals [27]. Therefore, the apparent hydrochromic behavior is not a consequence of a dehydration-induced ET, i.e., electrochromism typical for extensively studied viologens, which can undergo reversible one-electron reduction and form stabile, vividly colored, radical cations [9].

The results of the IR spectral study are in accordance with the results above showing the predomination of charge-transfer transitions between HCF and TOXO on the vibrational time scale at RT [8] (Figure S4). The in situ, time-dependent ATR study (Figure 4b) undoubtably proves the role of water in the reversible hydrochromic character of **1a** and **1b**.

The effect of the dehydration of **1a** disrupts the site symmetry to the extent that one *ν*(C≡N)_cyano_ band in the spectrum of **1a** splits into two *ν*(C≡N)_cyano_ bands, observed in the spectrum of **1b**. This reveals the existence of two nonequivalent groups of CN^−^ ligands within the structure of **1b**, the first group involved in H-bonding and the second without any pertinent physical interactions. This is supported by the appearance of only one *ν*(C≡N)_cyano_ band in the spectrum of **1cr** (see Figure S4), which reflects the presence of equivalent CN^−^ ligands, all involved in H-bonding, as confirmed from the crystal structure. Broad absorption *ν*(OH) bands in the 3250–3600 cm^−1^ region, along with a well-defined *δ*(H-O-H) vibration at 1615 cm^–1^ within the spectrum of **1a**, characteristic of H-bonded water molecules, clearly disappear during the transformation of **1a** to **1b**. The skeletal vibrations in HCF, i.e., *ν*(CN), *ν*(Fe-C), and *δ*(Fe-C≡N), and in TOXO, i.e., *ν*(C=N)_oxime_, *ν*(CC, CN)_pyridinium ring_ and *ν*(NO)_oxime_, are preserved with only small shifts relative to their initial absorption frequencies (for details see Figure S4 in Supplemental Material).

**Figure 5:**
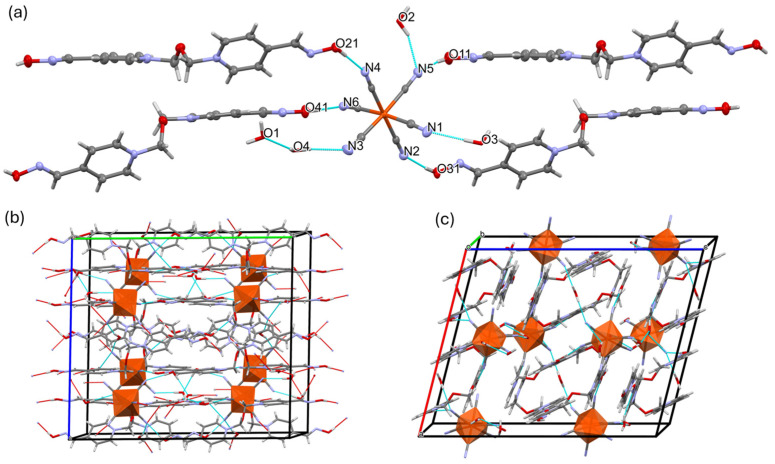
(**a**) Hydrogen bonding motif around the HCF core; (**b**) 3D supramolecular network viewed along the a-axis; (**c**) Ccystal packing viewed along the b-axis.

**Structure description of 1cr.** The crystal structure of monoclinic (TOXO)_2_[Fe(CN)_6_]·3.5H_2_O (**1cr**) can be described as a supramolecular 3D assembly consisting of [Fe(CN)_6_]^4–^ octahedra surrounded by four protonated TOXO cations and four water molecules. The observed Fe-C bond distances, ranging from 1.898(2) to 1.931(2) Å, are consistent with the iron divalent oxidation state and with the Fe-C bond lengths in crystal structures of supramolecular HCF-based complexes deposited in CSD, which are in the range 1.886–1.947 Å. The C≡N bond lenghts are all similar, ranging from 1.155(3) to 1.162(3). The octahedron around iron can be considered as slightly distorted, with C-Fe-C angles ranging from 86.48(9) to 93.61(9)°. The supramolecular motif around the HCF core, shown in Figure 5a, is established by the formation of four H-bonds between nitrogen atoms from the CN^−^ ligands (N2, N4, N5, and N6) and hydrogen atoms from the oxime groups (O11, O21, O31, and O41). The shortest H-bonds are O_oxime_-H···N_cyano_ (2.633(2)–2.702(2) Å). The nitrogen atom N5 also forms an additional H-bond with the water molecule O2. The remaining two cyano nitrogen atoms (N1 and N3) are H-bonded to water molecules (O3 and O4). Moreover, the water molecule O4 extends H-bonding towards the second water molecule O1. It is important to highlight the interconnectivity of all water molecules (except O2), which is the feature leading to the formation of a supramolecular network that extends in all three dimensions, as shown in Figure 5b. In addition, each TOXO cation in this 3D assembly, containing two oxime groups H-bonded to two HCF cores, is a supramolecular bridging species acting as a pillar. The crystal data and asymmetric unit for **1cr**, comprising two protonated TOXO cations, one HCF anion, and 3.5 water molecules, are shown in Table S1 and Figure S5.

**Figure 6:**
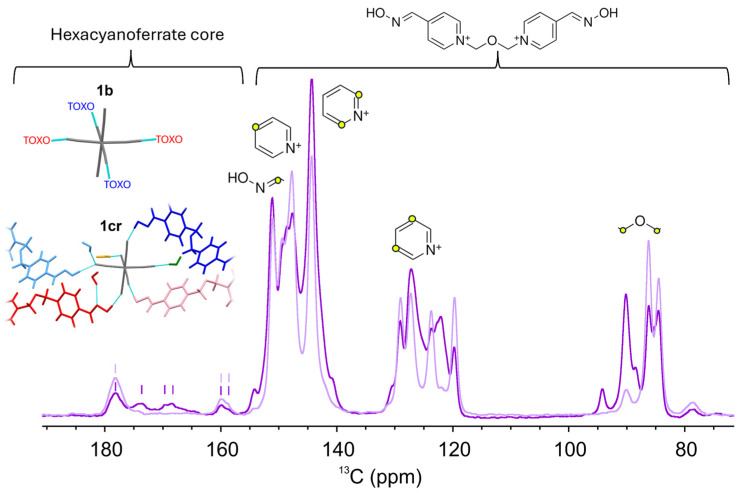
^13^C MAS NMR spectra of **1cr** (dark violet) and **1b** (violet) obtained with a sample spinning frequency of 10 kHz.

**Solid-state NMR study.** Further insight into the structural features of **1b** was obtained by the comparative analysis of the solid-state ^13^C MAS NMR spectra of **1cr** and **1b** (Figure 6). To the best of our knowledge, a solid-state NMR analysis of such supramolecular hybrid donor–acceptor organic HCF complexes has never been reported. Interpretation of the ^13^C MAS NMR spectrum of **1cr** was conducted considering the crystal structure of **1cr**, as determined by the X-ray diffraction, which furthermore enabled elucidation of ^13^C MAS NMR signals in the case of **1b**. The spectra of both complexes comprise two clearly distinguishable parts: one with a set of ^13^C signals corresponding to C-atoms of TOXO cations between 80 and 155 ppm, and the other characteristic of CN^−^ ligands within HCF between 155 and 180 ppm. The observed sets of signals are identified (Table S2) on the basis of known ^13^C NMR (DMSO-d_6_) chemical shifts for monopyridinium derivatives [28], solid-state NMR analyses of dipyridinium carboxylate derivatives [29], and potassium hexacyanoferrate(II) trihydrate [30].

In the ^13^C MAS NMR spectrum of **1cr**, six signals, belonging to cyano carbon atoms, are observed, which is in accordance with the determined crystal structure of **1cr**, where four CN^−^ groups are H-bonded by four nonequivalent TOXO cations and/or TOXO–water assemblies (colored in red, light red, blue, and light blue in Figure 6, inset), while the remaining two cyano groups are H-bonded to two crystallographically independent water molecules (colored in green and orange in Figure 6, inset). On the other hand, the ^13^C MAS NMR spectrum of **1b** shows only three signals belonging to cyano carbon atoms: two signals can be ascribed to the presence of two pairs of nonequivalent TOXO cations (colored in red and blue in Figure 6, inset), while the third signal likely belongs to two identical CN^−^ ligands that remain free of any H-bond interactions. Such interpretation is in accordance with IR spectral analysis of **1b**. The existence of free CN^−^ ligands strongly suggests that **1b** crystallizes in the form of a 2D layered structure with hydrophilic voids or channels, that allows fast intake of water molecules upon exposure to humidity, consequently leading to its hydrochromic transformation to **1a**.

### 2.2. Inter-Ionic Pentacyanonitrosylferrate Supramolecular Complex

The self-assembly between the photochromic NP anion and the TOXO cation in water results in spontaneous formation of a new insoluble, orange, crystalline hybrid salt, (TOXO)[Fe(CN)_5_(NO)]·2H_2_O (**2**).

**Figure 7:**
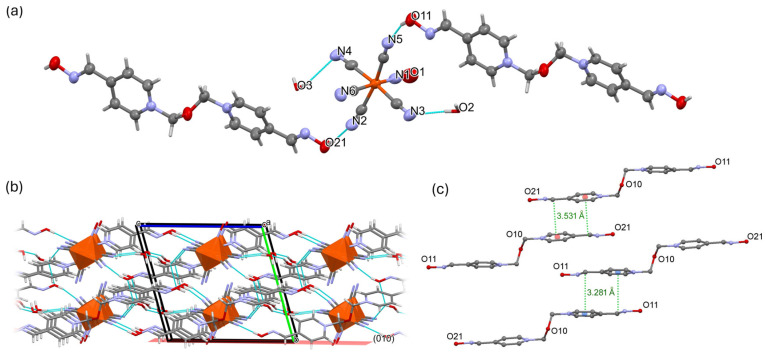
(**a**) Hydrogen bonding motif around the pentacyanonitrosylferrate core; (**b**) 2D supramolecular layers parallel to (010); (**c**) π···π interaction of pyridinium ring and oxime group with perpendicular distances of the centroids Cg of rings and to the planes through their centrosymmetrically related rings.

**Structure description of 2.** The crystal structure of triclinic TOXO[Fe(CN)_5_(NO)]·2H_2_O (**2**) exhibits a 2D layered assembly consisting of [Fe(CN)_5_(NO)]^2–^ octahedra surrounded by two protonated TOXO cations and two water molecules. The Fe-C distances are in the range 1.927(3) to 1.944(3) Å, while the Fe-N bond distance is significantly shorter, 1.655(3) Å. Also, the N≡O bond length (1.138(3) Å) is shorter than C≡N (1.139(3)–1.153(3) Å). These values are in good agreement with other polymeric structures reported for NP-based materials and the Fe-N and N-O distances are consistent with a multiple bond order in the linear FeNO [11,31,32]. Obviously, the NP octahedron is distorted, with the C-Fe-C/N angles ranging from 83.78(11) to 96.10(12)°. The greater distortion of the NP octahedron in comparison to HCF can also be seen from the sum of the deviations of 12 *cis* ligand-metal-ligand angles from 90°, which amounts to 28.2° in **1cr** and 43.05° in **2**. The supramolecular motif around the NP core (Figure 7a) consists of two short H-bonds (2.697(3) and 2.749(3) Å) formed between nitrogen atoms from two CN^−^ ligands (N2 and N5) and hydrogen atoms from oxime groups of two TOXO cations (O11, O21). Additionally, two cyano nitrogens (N3 and N4) form long H-bonds (around 3 Å) with two water molecules (O2 and O3). Such an H-bonding framework links the TOXO, NP, and water into layers parallel to (010) and creates alternating layers of TOXO and water molecules that are H-bonded to NP parallel to (1–10)/(-110) (Figure S6). The remaining cyano nitrogen (N6) and nitrosyl group do not participate in any supramolecular interactions. The 2D supramolecular layers parallel to (010) are shown in Figure 7b. Furthermore, parallel-displaced π···π interactions between the pyridinium ring of one TOXO and the oxime group of its centrosymmetrically related pair (Figure 7c) are identified. The perpendicular distances of the centroids Cg(ring N11-C15) and Cg(ring N21-C25) to the planes through their centrosymmetrically related rings are 3.281 and 3.531 Å, respectively. Such π···π interactions were also found previously within the supramolecular structure build up from HCF and *N*-methylpyridinium-4-oxime [8], revealing the propensity of the oxime group to create such interactions. The crystal data and asymmetric unit for **2,** containing one protonated TOXO cation, one NP, and two water molecules, are shown in Table S3 and Figure S7. The crystal structures of the supramolecular HCF-based **1cr** and NP-based **2** indicate that the decrease in the number of CN^−^ groups involved in H-bonding from six to four causes a reduction in the network dimensionality from 3D found in **1cr** to 2D in the case of **2**.

**Figure 8:**
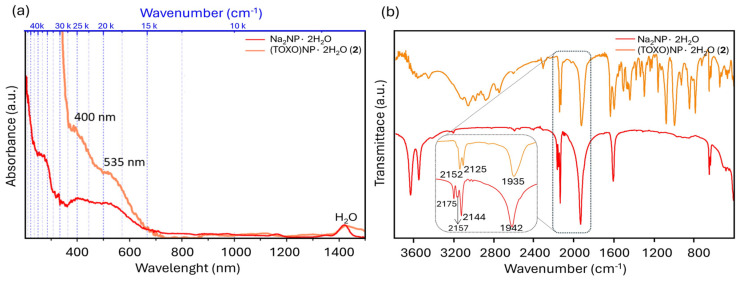
(**a**) UV–vis–NIR diffuse reflectance spectrum of **2** compared to sodium nitroprusside; (**b**) IR spectrum of TOXO-NP·2H_2_O (**2**) in correlation with Na-NP·2H_2_O at RT.

The electronic structure for NP is dominated by the nitrosyl group, leading to disfavored π-back donation from the iron toward the CN^−^ ligands when compared with that expected for HCF [11]. Consequently, in HCF and other transition metal cyanides, the electron density on the N-atom of the CN^−^ ligand is much more pronounced than in NP [31]. To determine whether TOXO and NP are forming a donor–acceptor inter-ionic CT complex, the UV–vis–NIR diffuse reflectance spectra of both **2** and sodium nitroprusside are recorded (Figure 8a). The spectrum of NP is characterized by the occurrence of six bands resulting from intra-molecular metal-to-ligand charge transfer (MLCT) from the Fe center to the coordinated CN^−^ and NO^+^ ligands [11]. The UV MLCT bands around 210 nm and 260 nm in sodium nitroprusside are covered in the spectrum of **2,** with the strong absorption bands of the TOXO cation itself. In the visible spectral region, two MLCT bands dominate in both spectra at ~400 nm and ~535 nm, corresponding to the transitions from the two Fe-character orbitals to the π*NO orbital within the NP core, in accordance with data in the literature [11]. This explains the orange color of **2** being like sodium nitroprusside itself. The band in the NIR region is assigned to water molecules. Evidently, TOXO in interaction with NP is an electronically “innocent” cation, acting as a charge-compensating counterion.

The results of the diffuse reflectance spectroscopy agree with the IR analysis (Figure 8b), showing the lack of driving force for the CT and ET processes within the vibrational time scale at RT. The two well-resolved *ν*(CN) bands of similar intensities at 2152 cm^−1^ and 2125 cm^−1^ are associated with nonequivalent CN^−^ ligands. These bands are assigned to four CN^−^ ligands involved in H-bonding and one CN^−^ ligand uninvolved in any pertinent physical interaction, which is in accordance with the established formation of the 2D layered solid. More detailed assignation of the IR bands for **2** is given in the Supplemental Materials. The ^57^Fe Mössbauer spectra of **2** at RT and 80 K exhibit small isomer shifts (IS~−0.21 mm s^−1^) and large quadrupole splitting (QS~1.85 mm s^−1^), as shown in Figure S8. Like in other nitroprussides, an axial deformation of the coordination sphere of the iron is observed as a temperature-independent large quadrupole splitting, while domination of the nitrosyl group, which forms a strong σ bond with the iron atom accompanied by a large charge subtraction from the latter via π-back donation, explains the extremely low values of the isomer shift [11,32]. The strongly bonded CN^–^ and NO^+^ ligands in the structural {Fe(CN)_5_NO} unit effectively shield the iron, which adopts a low-spin electronic configuration and is practically independent from changes in the outer environment, as was established for coordination NP polymers [32,33]. The presented results undoubtedly emphasize the general differences in the electronic properties between NP and HCF. In the IR spectrum of **2,** and in the two phases **1cr** and **1b**, the *ν*(CN) vibration is found with a frequency difference of about 80 cm^–1^ between equivalent vibrational modes, while the difference in the isomer shifts between **2** and **1b** is about 0.19 mm s^–1^. Interestingly, there is a pronounced analogy between the properties found for these supramolecular polymer structures and the low-spin cyanoiron coordination polymers [11,33,34,35]. The HCF unit has a better electron-donor ability than NP, which is supported in the presented interactions with the TOXO, containing two pyridinium oxime units, with its pronounced electron-acceptor ability.

## 3. Materials and Methods

### 3.1. Chemicals and Experimental Techniques

Toxogonin^®^ (Obidoxime chloride, TOXO-2Cl; Merck KGaA, Darmstadt, Germany), potassium hexacyanoferrate(II) trihydrate (K_4_[Fe(CN)_6_]·3H_2_O; Merck KGaA, Sigma-Aldrich solutions, Darmstadt, Germany), sodium nitroprusside dihydrate (Na_2_[Fe(CN)_5_(NO)]·2H_2_O; Merck KGaA, Sigma-Aldrich solutions, Darmstadt, Germany), and potassium chloride (Merck KGaA, Sigma-Aldrich solutions, Darmstadt, Germany) were reagent-grade chemicals and were used as purchased. Ultra-pure water (ASTM Type 1 quality), obtained using a Millipore Direct-Q 5 purification system (Merck KGaA, Darmstadt, Germany) was used to prepare solutions.

Elemental analysis was performed using a Perkin Elmer 2400 Series II CHNS elemental analyzer (PerkinElmer, Waltham, MA, USA) with an accuracy of ±0.3%.

FTIR spectra were recorded at room temperature in the range 4000–400 cm^–1^ using a PerkinElmer Spectrum Two FTIR spectrometer (PerkinElmer, Waltham, MA, USA) equipped with a diamond UATR accessory and a pressure arm with a force indicator.

The thermogravimetric and differential thermal analyses were performed using a Shimadzu DTG-60H instrument (Shimadzu Corp., Kyoto, Japan) in a stream of N_2_ (50 mL/min) at a heating rate of 10 °C/min. The differential scanning calorimetry was performed using a STA 449 F5 Jupiter (Erich Netzsch B.V. & Co. Holding KG, Selb, Germany).

The Mössbauer spectra were recorded at 298 K and at 80 K in the transmission mode using a conventional WissEL spectrometer (W5) (WissEL GMBH, Starnberg, Germany). The 10 mCi activity ^57^Co in a rhodium matrix was used as the Mössbauer source. Liquid He cryostat (JANIS) was used at 80 ± 0.2 K temperature measurements. The velocity calibration was made using metallic α-Fe absorber at 25 °C. Quantitative analyses of the Mössbauer spectra were performed using the MossWin program [36].

The X-ray powder diffraction patterns recorded at RT and the patterns recorded at 100 °C during temperature-induced in situ monitoring by means of high-temperature X-ray powder diffraction (HT-XRPD) were obtained using a Philips MPD 1880 counter diffractometer (Philips/Malvern Panalytical Ltd., Malvern, UK) with CuK_α_ radiation equipped with an Anton Paar high-temperature chamber. The diffraction data were collected in the 2θ range 6–36° with a step of 0.02° and fixed counting time of 5 s per step.

Solid-state ^13^C NMR experiments were performed on a Bruker AVANCE NEO 400 MHz NMR spectrometer (Bruker, Ettlingen, Germany) using a 4 mm CP-MAS probe. The sample spinning frequency was 10 kHz. The ^13^C CP-MAS NMR experiments consisted of excitation of protons with a p/2 pulse of 3.5 μs, CP block of 2 ms, and signal acquisition with high-power proton decoupling. A total of 13,312 scans was accumulated with a repetition delay of 3 s. The chemical shifts were referenced externally using adamantine. The spectra were measured at 25 °C. All spectra were analyzed using the Bruker TopSpin 4.2 software.

Diffuse reflectance spectra were recorded at room temperature in the range 300–2000 nm in both the reflectance and absorbance modes using a Shimadzu UV-3600 spectrophotometer (Shimadzu Corp., Kyoto, Japan) equipped with an integrated sphere and calibrated against a surface of barium sulfate for 100% reflectance. The sample preparation was performed as follows: the barium sulfate was placed in the holder and the pure sample, previously ground to fine powder, was carefully adsorbed to the central position of the barium sulfate surface. The optical band gap energies (*E*_g_) were determined as the intersection point between the energy axis and the line extrapolated from the linear portion of the absorption edge in a plot of (F(*R*)h*υ*)^2^ against energy E. The Kubelka–Munk function, *F* = (1 – R)^2^/2R, was converted from the recorded diffuse reflectance data, where *R* is the reflectance of an infinitely thick layer at a given wavelength.

The high-resolution scanning electron microscope Apreo 2S (ThermoFisher Scientific Inc., Waltham, MA, USA) was used to obtain an HR-SEM image of the sample. A scanning transmission electron microscopy (STEM) analysis was conducted on a state-of-the-art Jeol ARM 200 CF STEM instrument (JEOL Ltd., Tokyo, Japan). The STEM was equipped with a cold field-emission gun (FEG) and a Jeol Centurio energy-dispersive X-ray spectroscopy (EDXS) system with a 100 mm^2^ SDD detector and a Gatan GIF Quantum ER dual electron energy loss spectroscopy (EELS) system. The sample was transferred to a lacey-carbon-coated Cu TEM grid and investigated in the probe Cs-corrected STEM at 80 kV of accelerating voltage to minimize potential damage to the sample due to electron irradiation.

### 3.2. Isolation of Supramolecular Complexes

In general, (TOXO)_2_[Fe(CN)_6_]·nH_2_O solids were self-assembled and precipitated from aqueous solutions containing the TOXO dication and hexacyanoferrate(II) anion, [Fe(CN)_6_]^4–^, in a 2:1 molar ratio, or hexacyanoferric acid, H_4_[Fe(CN)_6_], in a 1:1 molar ratio. Once precipitated from aqueous solution, the solids could not be re-dissolved in water or in common polar organic solvents such as MeOH, EtOH, acetone, and DMSO. The H_4_[Fe(CN)_6_] was synthetized following the procedure described in [22].

(TOXO)_2_[Fe(CN)_6_]·8H_2_O (**1a**): The as-prepared reddish brown product instantaneously precipitated at RT by the addition of an aqueous solution of TOXO-2Cl (86.2 mg; 0.24 mmol in 4.0 mL) to an aqueous solution of K_4_[Fe(CN)_6_]·3H_2_O (50.6 mg; 0.12 mmol in 3.0 mL). The precipitate was filtered off, washed with water, dried within the fold of filter paper to remove moisture, and then stored in a desiccator saturated with wet air. The mass of the complex was 94.1 mg (η = 84.1%). A thermogravimetric analysis was performed in the temperature range 25–160 °C. The calculated mass loss for 8 H_2_O was 15.45%, while the observed mass loss was 15.90%. FTIR (UATR, cm^−1^): *ν*(OH)_water_, 3365 (br); *ν*(CN)_cyano_, 2040 (vs); *ν*(C=N)_oxime_, 1639 (s); *ν*(CC, CN)_pyridinium ring_, 1614 (s), 1569 (w), 1514 (m); *ν*(C–O–C)_linker_, 1090 (s); *ν*(N–O)_oxime_, 1010 (s); *δ*(Fe–C), 581 (m); *ν*(Fe-C), 429 (m) cm^–1^.

(TOXO)_2_[Fe(CN)_6_] (**1b**): The as-prepared **1a** transformed to **1b** by extrication of water molecules induced by washing the precipitate with absolute EtOH and then drying it in the desiccator under reduced pressure over P_4_O_10_ for 24 h. The isolation procedure and spectroscopic analysis of **1b** were reported in our previous work [8]. The purity of **1b** used in this work was confirmed by elemental and thermal analyses. Elemental analysis calculated (%) for C_34_H_32_N_14_O_6_Fe (*M*_r_ = 788.57): C 51.79, H 4.09, N 24.87; found: C 51.90, H 3.87, N 24.44. EDXS iron analysis: Fe 7.1; found: Fe 7.2. Thermogravimetric analysis in the temperature range 25–160 °C: no significant weight loss was observed.

(TOXO)_2_[Fe(CN)_6_]·3.5H_2_O (**1cr**): The TOXO-2Cl (86.2 mg; 0.24 mmol) was dissolved in 4.0 mL of 3.0 M KCl solution and added to the aqueous solution of K_4_[Fe(CN)_6_]·3H_2_O (50.6 mg; 0.12 mmol in 3 mL of 1.0 M KCl). The clear pale yellow mixture was left in the dark at 4 °C for 48 h. The dark violet SCXRD-quality crystals were formed, filtered off, washed with water, and dried under reduced pressure. The mass of the complex was 52.3 mg (η = 51.2%). Elemental analysis calculated (%) for C_34_H_39_N_14_O_9.5_Fe (*M*_r_ = 851.62): C 47.95, H 4.61, N 23.03; found: C 48.06, H 4.73, N 23.32. Thermogravimetric analysis in the temperature range 25–160 °C: calculated weight loss for 3.5 H_2_O: 7.40%; observed mass loss: 7.30%. FTIR (UATR, cm^−1^): *ν*(OH)_water_, 3396 (br); *ν*(CN)_cyano_, 2038 (vs); *ν*(C=N)_oxime_, 1640 (s); *ν*(CC, CN)_pyridinium ring_, 1613 (s), 1515 (m); *ν*(C–O–C)_linker_, 1093 (s); *ν*(N–O), 1012 (s); *δ*(Fe–C), 580 (m); *ν*(Fe-C), 428 (m) cm^–1^.

(TOXO)[Fe(CN)_5_(NO)]·2H_2_O (**2**): The TOXO-2Cl (46.7 mg; 0.13 mmol) and the Na_2_[Fe(CN)_5_(NO)]·2H_2_O (38.7 mg; 0.13 mmol) were dissolved in 2.0 mL of water in separate beakers and the solutions were mixed under stirring. The mixture was left in darkness at RT, resulting in the formation of an orange crystalline precipitate. The precipitate was filtered off, washed several times with deionized water, and dried in the desiccator under reduced pressure over P_4_O_10_ for 24 h. Elemental analysis calculated (%) for C_19_H_20_N_10_O_6_Fe (*M*_r_ = 540.28): C 42.23, H 3.73, N 25.92; found: C 42.43, H 3.38, N 25.52. A thermogravimetric analysis was performed in the temperature range 25–160 °C. The calculated mass loss for 2 H2O was 6.69%, while the observed mass loss was 6.77%. FTIR (UATR, cm^−1^): *ν*(OH)_water_, 3562 (br,s); *ν*(OH)_water_ + *ν*(OH)_oxime_ 3452 (m); *ν*(CN)_cyano_, 2152 (s), 2130 (s); *ν*(NO)_nitrosyl_, 1935 (vs) cm^−1^; *ν*(C=N)_oxime_, 1640 (vs); *ν*(CC, CN)_pyridinium ring_, 1618 (vs), 1515 (m); *δ*(HOH)+ *ν*(CC, CN)_pyridinium ring_1618 (vs); *ν*(C–O–C)_linker_, 1093 (s); *ν*(NO)_oxime_, 1010 (s); *δ*(Fe-NO_nitrosyl_), 666 (s); *δ*(Fe-C≡N), 647 (s); *ν*(Fe–C), 410 (m-s). The SCXRD-quality crystals were obtained by a similar procedure by using the ethanolic solution of Na_2_[Fe(CN)_5_(NO)]·2H_2_O and leaving the mixture in the dark at 4 °C for 48 h. An XRPD pattern of the prepared crystals was in good agreement with that calculated from the single-crystal structure, as shown in Figure S9.

### 3.3. SCXRD Structure Determination

Diffraction data were collected from suitable single crystals of **1cr** and **2** on an Oxford Diffraction Xcalibur single-crystal diffractometer with an Xcalibur Sapphire 3 CCD detector and Mo Ka radiation. The crystals were glued to thin glass needles. A crystal of **1cr** was transferred into a stream of cold nitrogen and data were collected at 150 K, while those for **2** were collected at room temperature. The CrysAlis Pro Software system, version 1.171.37.35 was used for data collection and reduction (CrysAlisPro, Agilent Technologies, Version 1.171.37.35, release 13-08-2014 CrysAlis171 .NET). The X-ray diffraction data were corrected for the Lorentz-polarization factor, and absorption effects by the multi-scan method empirical absorption correction using spherical harmonics implemented in SCALE3 ABSPACK scaling algorithm. The Olex2 package was used for structure solving and refinement [37]. The structures were solved with the SHELXT [38] structure solution program using intrinsic phasing and refined with the SHELXL [39] refinement package using least-squares minimization based on F^2^ against all reflections. All non-hydrogen atoms were refined anisotropically. Hydrogen atoms bound to carbon atoms were placed in calculated positions and refined using the riding model, with Uiso(H) values set at 1.2Ueq of their carbon atom. Hydroxyl hydrogen atoms were refined by using the rotating group refinement (HFIX 147) with Uiso(H) values set at 1.5Ueq of the oxygen atom. Water hydrogen atoms had fixed geometry (AFIX 6). The geometry parameters were calculated using Olex. The structure drawings were prepared using MERCURY [40].

The crystallographic data are summarized in Tables S1 and S3 in the Supplemental Materials. CCDC 2314251 and 2314252 contain the supplementary crystallographic data for this paper. These data can be obtained free of charge from The Cambridge Crystallographic Data Centre via www.ccdc.cam.ac.uk/structures (accessed on 19 March 2024).

## 4. Conclusions

We have characterized the structural and electronic features of supramolecular inter-ionic solids, self-assembled in aqueous media by the H-bonding association between the bis-pyridinium oxime dication, Toxogonin^®^ and the hexacyanoferrate(II) or nitroprusside anion. The electron-donor character of the hexacyanoferrate(II) ion led to the formation of donor–acceptor CT complexes, while in the case when the nitroprusside ion was used, the Toxogonin^®^ acts solely as a charge-compensating counterion. The formation of two hydrated phases of (TOXO)_2_[Fe(CN)_6_] revealed the importance of association rate control in the final supramolecular assembly. The hydrochromic phase, in contrast to single-crystal phase, showed a reversible color change induced by dehydration. The loss of water molecules enhances the CT interaction but without detectable electron transfer. The resolved crystal structures of the two complexes show the pronounced self-assembly potential of Toxogonin^®^ as a profound H-bond donor with the capability to form π···π interactions. In association with hexacyanoferrate(II), the 3D network is formed with Toxogonin^®^ as an H-bond bridging species acting as a pillar. In contrast, the nitroprusside ion governed the formation of the 2D layered structure, further stabilized by π···π interactions. Due to the widespread use of Toxogonin^®^ as a biologically important and therapeutic substance, we believe that assessing the properties dictated by its π-conjugated acceptor ability resulting in distinctive structural interrelations and the optical CT energies in the solid state represent an important contribution.

## Data Availability

Data are contained within the article and Supplementary Materials.

## Supplementary Materials

The following supporting information can be downloaded at: https://www.mdpi.com/article/10.3390/molecules29081698/s1, Figure S1: Thermal analysis trace for **1a**, **1b**, and **1cr** in a stream of nitrogen; Figure S2: UV–vis–NIR diffuse reflectance spectrum of **1cr**; Figure S3: ^57^Fe Mössbauer spectra of the **1b** relative to α-Fe at 80 K (left) and RT (right); Figure S4: IR spectra of TOXO-Cl, K-HCF ·3H_2_O, and related IICT-complexes TOXO-HCF·3.5 H_2_O, **1cr**, and anhydrous TOXO-HCF, **1b** at RT; Table S1: Crystal data and structure refinement for **1cr**; Figure S5: Asymmetric unit of **1cr** with the atom labeling scheme. Ellipsoids are at the 30% probability level; Table S2: ^13^C solid-state NMR chemical shifts for **1cr** and **1b** at RT; Figure S6: View of **2** along the *c*-axis. Two double layers are colored in red and blue; Table S3: Crystal data and structure refinement for **2**; Figure S7: Asymmetric unit of **2** with the atom-labeling scheme. Ellipsoids are at the 30% probability level; Assignation of bands in IR spectrum of TOXO-NP·2H_2_O (**2**) in correlation with Na-NP·2H_2_O at RT shown in Figure 8b; Figure S8: ^57^Fe Mössbauer spectra of **2** relative to α-Fe at 80 K (left) and RT (right). Figure S9. XRPD patterns of **2** measured and simulated from the crystal structure.
