# Supplementary material for: Supramolecular Solid Complexes between Bis-pyridinium-4-oxime and Distinctive Cyanoiron Platforms
Source: Molecules. 2024 Apr 9;29(8):1698. doi: 10.3390/molecules29081698 (PMC11052229; doi:10.3390/molecules29081698)
Supplement: Supplementary file 1 [file molecules-29-01698-s001.zip › molecules-2949999-supplementary.pdf]

Supramolecular Solid Complexes between bis(Pyridinium-4-oxime) and Distinctive Cyanoiron Platforms

Igor Picek [1], Dubravka Matković-Čalogović [2], Goran Dražić [3], Gregor Kapun [3], Primož Šket [3], Jasminka Popović [4,*] and Blaženka Foretić [1,*]

[1] Department of Chemistry and Biochemistry, School of Medicine, University of Zagreb, Šalata 3, HR-10000 Zagreb, Croatia; ipicek@mef.hr (I.P.); bforetic@mef.hr (B.F.);

[2] Department of Chemistry, Faculty of Science, University of Zagreb, Horvatovac 102a, HR-10000 Zagreb, Croatia; dubravka@chem.pmf.hr (D.M.Č.);

[3] National Institute of Chemistry, Hajdrihova 19, SLO-1001 Ljubljana, Slovenia; goran.drazic@ki.si (G.D.); gregor.kapun@ki.si (G.K.); primoz.sket@ki.si (P.S.)

[4] Division of Materials Physics, Ruđer Bošković Institute, Bijenička cesta 54, HR-10000 Zagreb, Croatia; Jasminka.Popovic@irb.hr (J.P.)

*Correspondence: bforetic@mef.hr (B.F.); Jasminka.Popovic@irb.hr (J.P.)

Contents:

Figure S1: Thermal analysis trace for **1a**, **1b** and **1cr** in the stream of nitrogen.

Figure S2: UV-Vis-NIR diffuse-reflectance spectrum of **1cr**.

Figure S3: ^{57}Fe Mössbauer spectra of the **1b** relative to α-Fe at 80 K (left) and RT (right).

Figure S4: IR spectra of TOXO-Cl, K-HCF ·3H_2O and related IICT-complexes TOXO-HCF·3.5 H_2O, **1cr**, and anhydrous TOXO-HCF, **1b** at RT.

Table S1: Crystal data and structure refinement for **1cr**.

Figure S5: Asymmetric unit of **1cr** with the atom labeling scheme. Ellipsoids are at the 30 % probability level.

Table S2: ^{13}C solid-state NMR chemical shifts for **1cr** and **1b** at RT.

Figure S6: View of **2** along the *c*-axis. Two double layers are colored in red and blue.

Table S3: Crystal data and structure refinement for **2**.

Figure S7: Asymmetric unit of **2** with the atom labeling scheme. Ellipsoids are at the 30 % probability level.

Assignation of bands in IR spectrum of TOXO-NP·2H_2O (**2**) in correlation with Na-NP·2H_2O at RT shown in Fig. 8b.

Figure S8: ^{57}Fe Mössbauer spectra of **2** relative to α-Fe at 80 K (left) and RT (right).

Figure S9: XRPD patterns of **2** measured and simulated from the crystal structure.

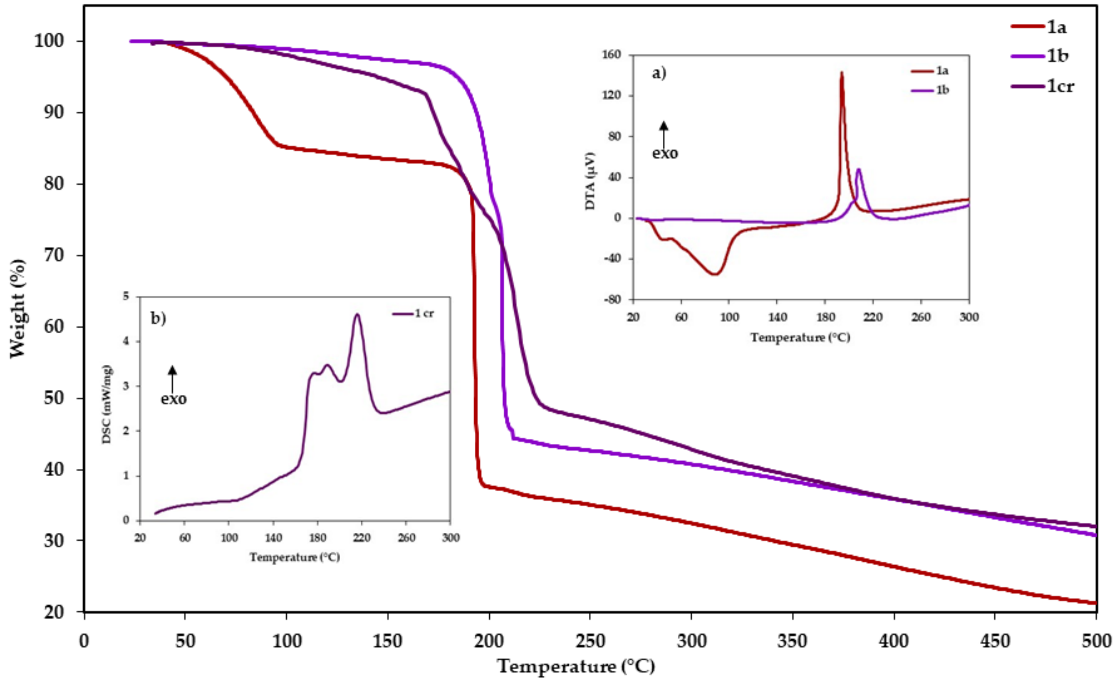

Figure S1. Thermal analysis trace for **1a**, **1b** and **1cr** in the stream of nitrogen.

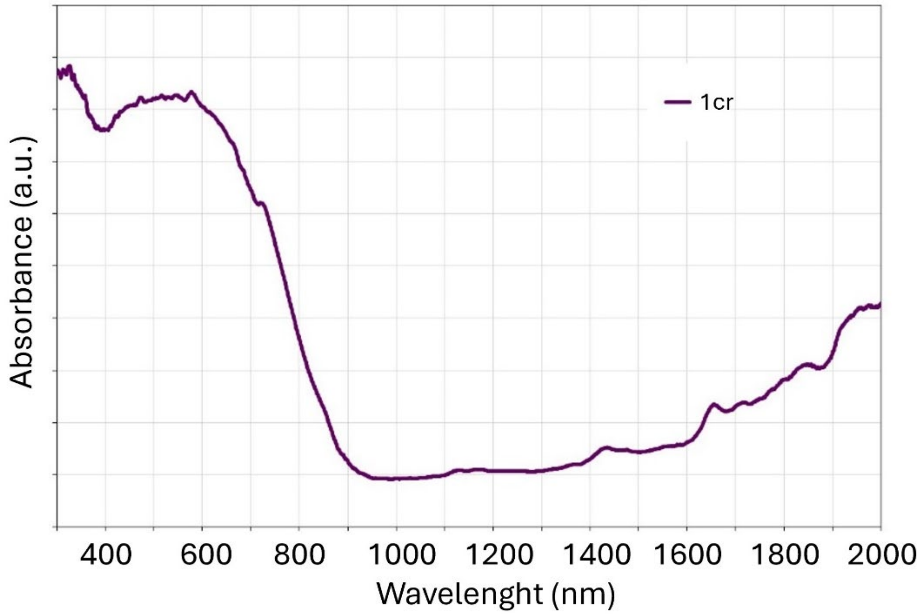

Figure S2. UV-Vis-NIR diffuse-reflectance spectrum of **1cr**.

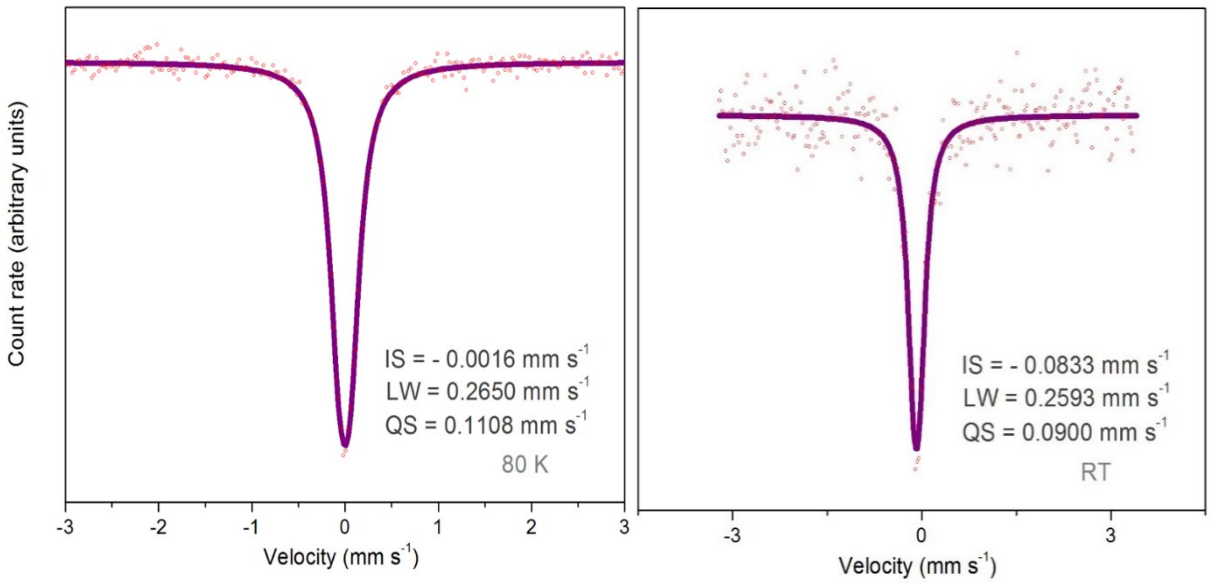

Figure S3. ^{57}Fe Mössbauer spectra of the **1b** relative to α-Fe at 80 K (left) and RT (right).

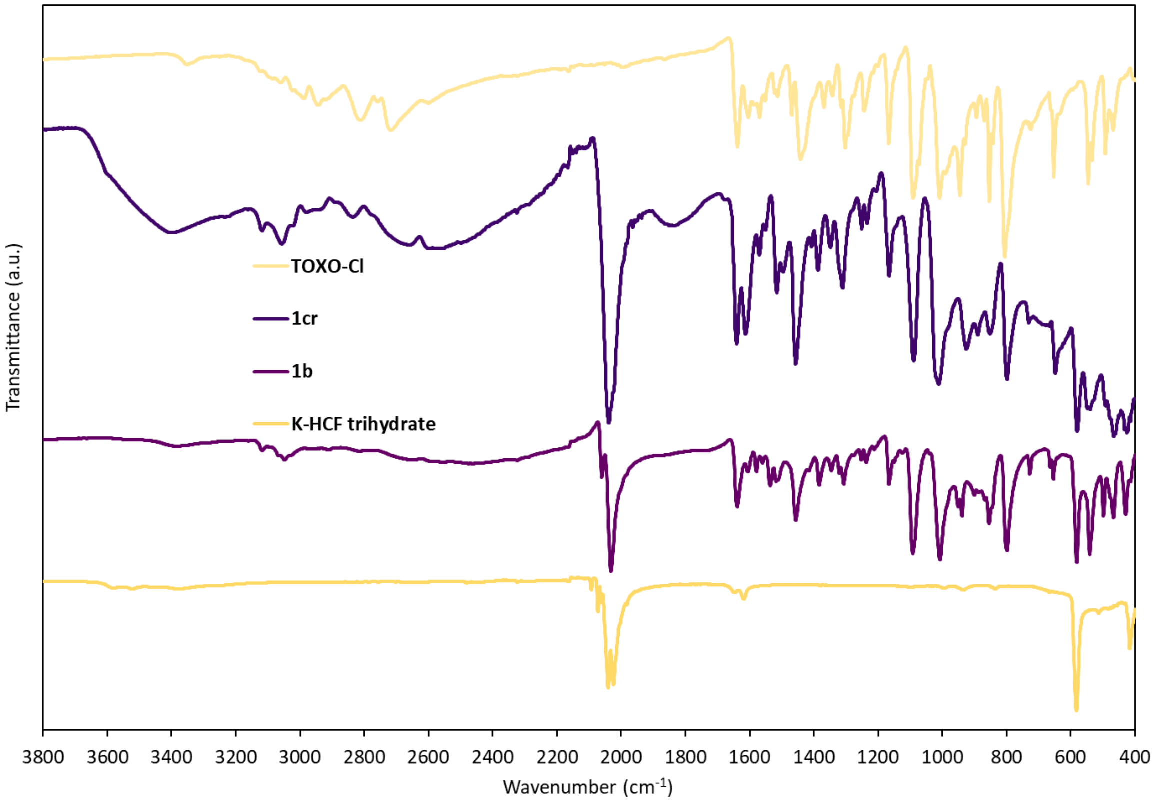

Figure S4. IR spectra of TOXO-Cl, K-HCF ·3H_2O and related IICT-complexes TOXO-HCF·3.5 H_2O, **1cr**, and anhydrous TOXO-HCF, **1b** at RT.

TOXO-Cl: ν(O-H)$_{oxime}$, 3440 (m, br) cm^{-1}; ν(C_{ring}-H)$_{pyridinium\ ring}$, $\nu_{sym,\ asym}$(CH_2)$_{linker}$ results in several medium and strong bands in the ~ 3300-2600 cm^{-1} region; the region ~ 1700–400 cm^{-1} contains mainly combination bands and the most prominent vibrations are ν(C=N)$_{oxime\ conjugated}$ at 1645 (vs) cm^{-1}, ν(CC, CN)$_{pyridinium\ ring}$ at 1608 (vs) cm^{-1} and 1515 (s) cm^{-1}, than ν(C–O–C)$_{linker}$ at 1089 (s) cm^{-1} and finally ν(NO)$_{oxime}$ at 1007 (vs) cm^{-1}.

K-HCF·3H_2O: broad and very weak ν(O-H)$_{water}$ absorption bands are in the 3350-3600 cm^{-1} region; in the ν(C≡N)$_{cyano}$ stretching region several peaks are observed, two of them are very strong bands at 2072 cm^{-1} and 2045 cm^{-1} in position and by number typically found in the potassium HCF salts; two weak but well-defined δ(H-O-H) vibrations probably corresponding to two nonequivalent water molecules, are at 1649 and 1619 cm^{-1} while strong bands at 583 and 420 cm^{-1}, respectively are result of δ(Fe–C≡N) bending and ν(Fe–CN) stretching.

TOXO-HCF·nH_2O (n=3.5 in **1cr**, and n=0 in **1b**): the skeletal vibrations in TOXO and HCF moieties, i.e. ν(C=N)$_{oxime}$ at ~1640 (vs) cm^{-1}, ν(CC, CN)$_{pyridinium\ ring}$ at 1608 (vs) cm^{-1} and 1515 (s) cm^{-1} and ν(NO)$_{oxime}$ at 999 (vs) cm^{-1}; ν(C≡N)$_{cyano}$ at 2038 (vs) cm^{-1} in **1cr** and in **1b** at

2060 (m) cm^{-1} and 2031(vs) cm^{-1}; δ(Fe-C≡N) at 580 (s) cm^{-1} and ν(Fe-C) at 429 (m) cm^{-1} are preserved with only small shifts relative to their initial absorption frequencies. In **1cr** waters have broad ν(O-H) absorption band centred around 3400 cm^{-1} and strong well-defined δ(H-O-H) vibration at 1619 cm^{-1}. In **1b** effect of dehydration disrupts the site symmetry to the extent that two $\nu(C\equiv N)_{cyano}$ bands appear.

Table S1. Crystal data and structure refinement for **1cr**.

Identification code	TOXO_HCF
Empirical formula	$C_{34}H_{39}N_{14}O_{9.5}Fe$
Formula weight	851.64
Temperature/K	150(1)
Crystal system	monoclinic
Space group	I2/a
a/Å	17.2565(5)
b/Å	22.4076(5)
c/Å	20.7624(6)

α/°	90
β/°	104.051(3)
γ/°	90
Volume/Å^3	7788.1(4)
Z	8
ϱ_{calc}g/cm^3	1.453
μ/mm^{-1}	0.461
F(000)	3544.0
Crystal size/mm^3	0.302 × 0.230 × 0.172
Radiation	Mo Kα (λ = 0.71073)
2Θ range for data collection/°	8.162 to 54.998
Index ranges	-21 ≤ h ≤ 22, -29 ≤ k ≤ 29, -20 ≤ l ≤ 26
Reflections collected	34143
Independent reflections	8899 [R_{int} = 0.0584, R_{sigma} = 0.0556]
Data/restraints/parameters	8899/1/544
Goodness-of-fit on F^2	1.015
Final R indexes [I>=2σ (I)]	R_1 = 0.0462, wR_2 = 0.0897
Final R indexes [all data]	R_1 = 0.0708, wR_2 = 0.0991
Largest diff. peak/hole / e Å^{-3}	0.27/-0.38

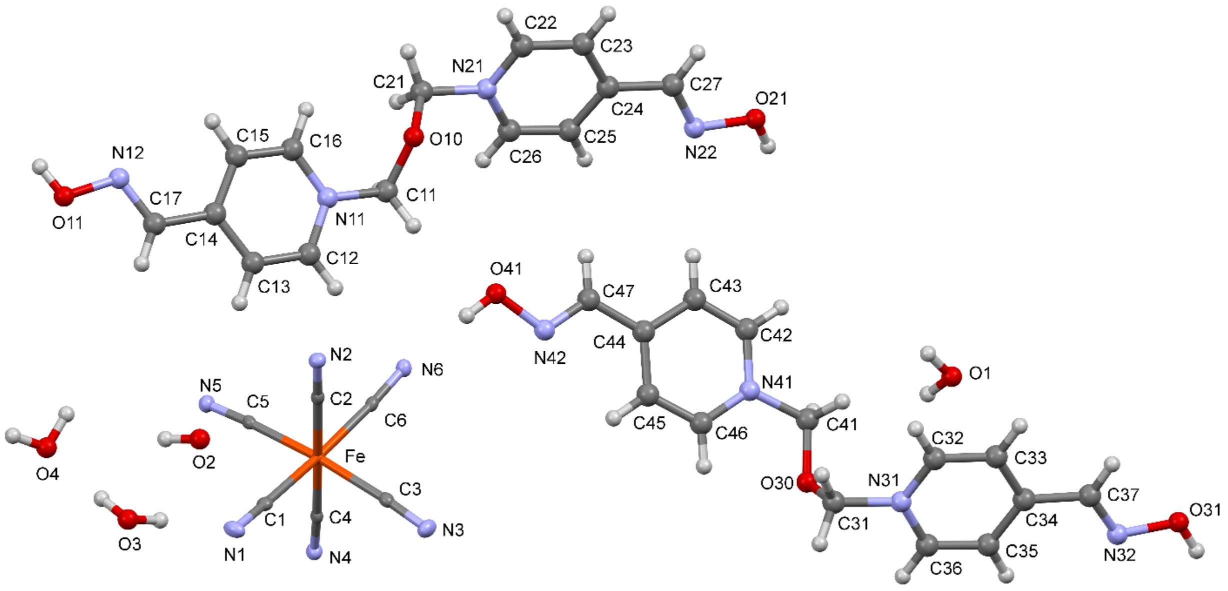

Figure S5. Asymmetric unit of **1cr** with the atom labeling scheme. Ellipsoids are at the 30 % probability level.

Table S2. ^{13}C solid-state NMR chemical shifts for **1cr** and **1b** at RT.

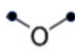		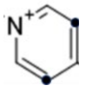		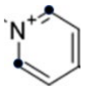		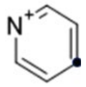		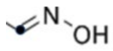		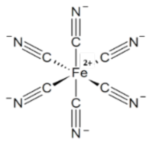	
δ_{iso}/ppm		δ_{iso}/ppm		δ_{iso}/ppm		δ_{iso}/ppm		δ_{iso}/ppm		δ_{iso}/ppm	
**1cr**	**1b**	**1cr**	**1b**	**1cr**	**1b**	**1cr**	**1b**	**1cr**	**1b**	**1cr**	**1b**
78.1	78.1	119.7	119.7	144.3	144.3	147.6	147.8	151.1	151.0	158.8	158.9
84.5	84.5	122.0	123.7							159.8	159.9
86.2	86.2	124.9	127.2							168.5	178.2
88.0	90.0	127.1	129.0							169.6	
90.1		129.1								173.8	
94.2		131.0								178.1	

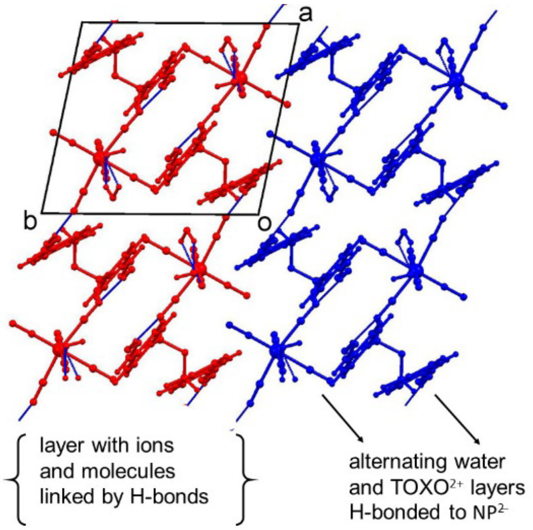

Figure S6. View of **2** along the *c*-axis. Two double layers are colored in red and blue.

Table S3. Crystal data and structure refinement for **2**.

Identification code	TOXO_NP
Empirical formula	$C_{19}H_{20}FeN_{10}O_6$
Formula weight	540.30
Temperature/K	293(2)
Crystal system	triclinic
Space group	P-1
a/Å	10.3296(10)
b/Å	11.0258(9)
c/Å	12.1832(11)
α/°	103.230(7)
β/°	110.695(9)

γ/°	95.886(7)
Volume/Å^3	1237.68(19)
Z	2
ρ_{calc}g/cm^3	1.450
μ/mm^{-1}	0.664
F(000)	556.0
Crystal size/mm^3	0.384 × 0.326 × 0.163
Radiation	Mo Kα (λ = 0.71073)
2Θ range for data collection/°	8.538 to 58
Index ranges	-11 ≤ h ≤ 14, -15 ≤ k ≤ 14, -16 ≤ l ≤ 11
Reflections collected	11207
Independent reflections	6328 [R_{int} = 0.0359, R_{sigma} = 0.0731]
Data/restraints/parameters	6328/0/333
Goodness-of-fit on F^2	0.995
Final R indexes [I>=2σ (I)]	R_1 = 0.0555, wR_2 = 0.1038
Final R indexes [all data]	R_1 = 0.1027, wR_2 = 0.1274
Largest diff. peak/hole / e Å^{-3}	0.37/-0.39

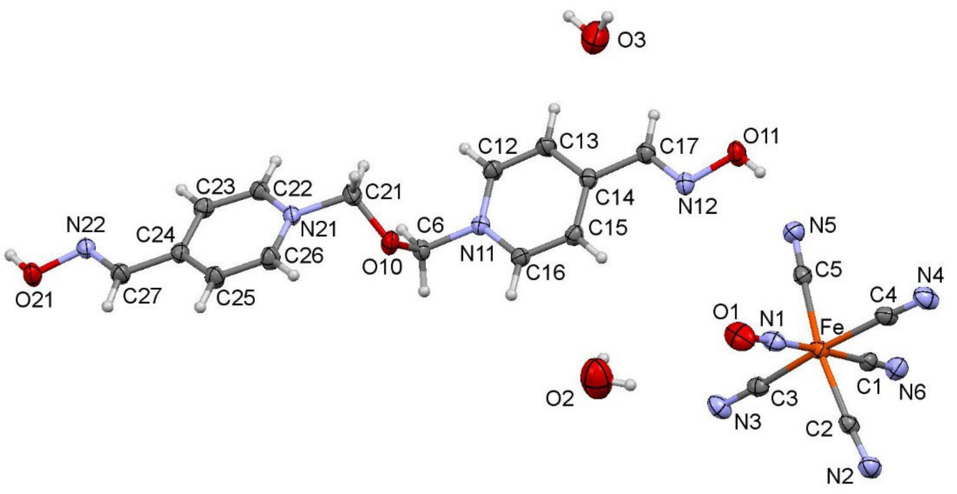

Figure S7. Asymmetric unit of **2** with the atom labeling scheme. Ellipsoids are at the 30 % probability level.

Assignation of bands in IR spectrum of TOXO-NP·2H$_2$O (2) in correlation with Na-NP·2H$_2$O at RT shown in Fig. 8b.

Na-NP·2H$_2$O: ν(O-H)$_{water}$ absorption bands at 3632 (vs) cm^{-1} and 3549 (vs) cm^{-1} (coordinated water); in the ν(C≡N)$_{cyano}$ stretching region absorption bands at 2175 (m) cm^{-1}, 2157 (m) cm^{-1} and 2144 (s) cm^{-1} are observed partially due to a slightly distorted coordination environment of iron and are followed by ν(NO)$_{nitrosyl}$ band at 1942 (vs) cm^{-1} (inset in Fig. 8b); the 1700–400 cm^{-1} region contains δ(HOH) band at 1619 (vs) cm^{-1} and δ(Fe-NO$_{nitrosyl}$) at 663 (s) cm^{-1}, than δ(Fe-C≡N) at 651 (s) cm^{-1} and ν(Fe–C) at 423 (m-s) cm^{-1}.

TOXO-NP·2H$_2$O: absorption bands at 3562 (br, s) cm^{-1} and 3452 (m) result from ν(OH)$_{water}$ vibration and the latter is combination ν(OH)$_{water}$ and ν(OH)$_{oxime}$, (broad absorption bands suggest hydrogen bonded water); two ν(CN)$_{cyano}$ absorption bands appear at 2152 (s-vs) cm^{-1} and lower energy 2125 (s-vs) cm^{-1} indicate, besides the distorted coordination environment, the possibility of existence of CN$^-$ ligand free from any pertinent electrostatic interactions (axial CN$^-$ ligand) and is followed with the vibration ν(NO)$_{nitrosyl}$ at 1935 (vs) cm^{-1} (inset in Fig. 8b); in the 1700–400 cm^{-1} region absorption band at 1640 (vs) cm^{-1} is due to ν(C=N)$_{oxime}$ while the band at 1618 (vs) cm^{-1} is a combination of ν(CN,CC)$_{pyridinium\ ring}$ and δ(HOH), followed by the ν(NO)$_{oxime}$ at 1010 (vs) cm^{-1} and finally the deformation and stretching bands involving the iron i.e. δ(Fe-NO$_{nitrosyl}$) at 666 (s) cm^{-1}, δ(Fe-C≡N) at 647 (s) cm^{-1} and ν(Fe–C) at 410 (m-s) cm^{-1}.

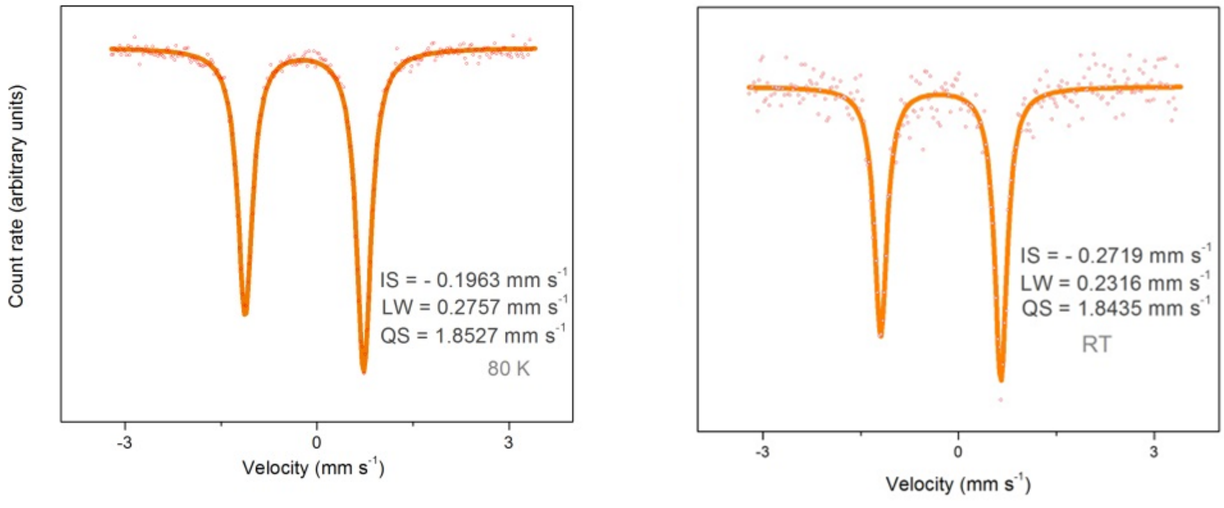

Figure S8. ^{57}Fe Mössbauer spectra of **2** relative to α-Fe at 80 K (left) and RT (right).

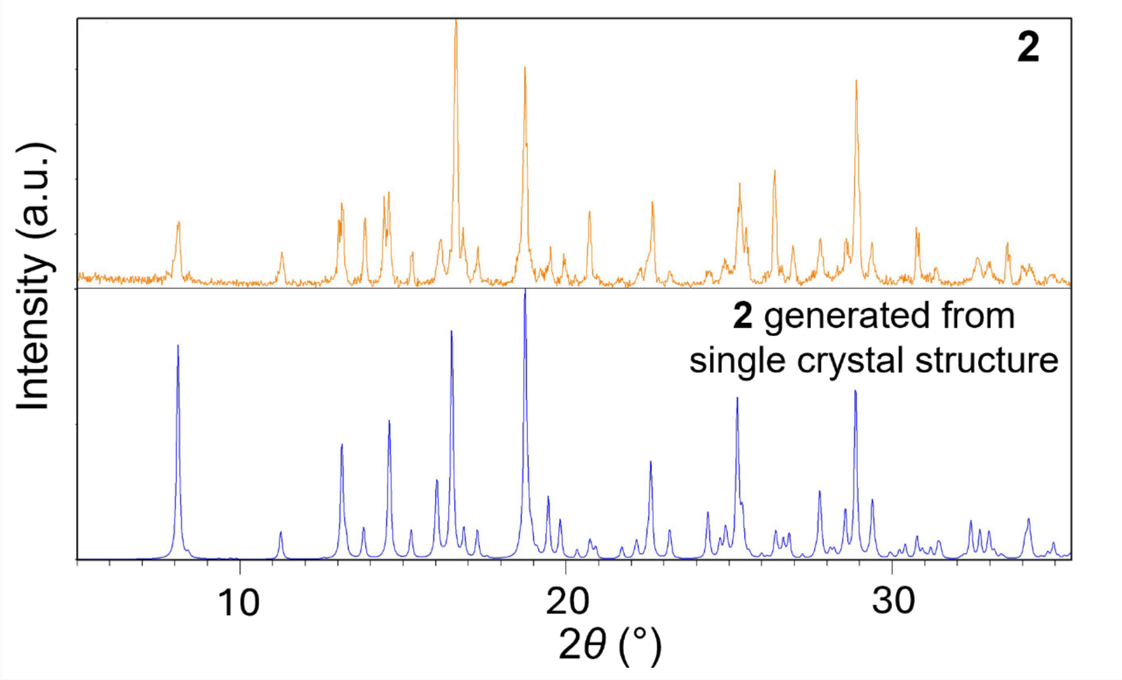

Figure S9. XRPD patterns of **2** measured and simulated from the crystal structure.
